# Australian teachers’ views on how primary science education can be improved

**DOI:** 10.1007/s13384-023-00638-4

**Published:** 2023-05-25

**Authors:** James Deehan, Amy MacDonald

**Affiliations:** Faculty of Arts and Education, School of Education, Charles Sturt University, Panorama Avenue, Bathurst, 2795 Australia

**Keywords:** Science education, Primary education, Teacher beliefs, Quality improvement

## Abstract

Teachers are crucial to bridging the theory-praxis divide in science education by utilising evidence-based teaching practices to improve outcomes for their learners. However, the perspectives of primary teachers have seldom been considered beyond the confines of specific professional development programs. This paper aims to explore Australian primary teachers’ beliefs about how primary science education could be improved. A sample of 165 primary educators responded to an open-ended digital survey prompt. The results showed that teachers viewed themselves and their colleagues as central to the improvement of primary science education as evidenced by the most prominent themes of Professional Development (47.27%), Funding-Resources (37.58%), Classroom Practice (21.82%) and Personal-Teacher Improvement (21.21%). Curiously, university did not feature strongly, suggesting the participants may hold neutral views regarding the impact of universities on primary science education. The findings should serve as a catalyst for future research and engagement with primary teachers. Universities could expand their roles in building relationships with and providing accessible professional development to a group of primary teachers who, quite rightly, view themselves as key to improving primary science education.

## Introduction and literature review

Science education is vital to our long-term prosperity as we collectively address global challenges, such as climate change, workforce changes, and technological advancements. Many nations are currently experiencing a fourth industrial revolution where complex digital skills and flexible work arrangements are creating information-based economies that threaten traditional routinised work (Burgess et al., 2021). Put simply, we need a global citizenry capable of utilising their science knowledge and skills in real-world settings (Bybee, 1997; Roberts & Bybee, 2014). This notion of scientific literacy is characterised by nonlinear thinking about science and its role in society (Hetherington et al., 2020; Roberts & Bybee, 2014). Foundational science education experiences play a significant role in establishing life-long science learning trajectories (Oates & Seah, 2021), as suboptimal science learning experiences in primary schooling can often worsen as students progress through secondary education (Ali et al., 2013; Denessen et al., 2015; DeWitt & Archer, 2015; Said et al., 2016). In fact, analysis has shown that despite a 16% increase in the total number of Australian students reaching year 12 between 1992 and 2012, the proportion of students completing a science subject declined by approximately 5% (Kennedy et al., 2014). More recent research suggests that these trends are not being reversed, with enrolment intentions and nonmetropolitan issues being particularly acute contemporary concerns (Kennedy et al., 2020; Murphy, 2022).

Of concern is that data from the Trends in International Mathematics and Science Study (TIMSS) indicate that Year 4 students, from Australia and other OECD nations, may be struggling in their scientific literacy development as over 90% of participating nations are falling below the high international threshold score of 550 (Martin et al., 1997; Thomson et al., 2020). Australia falls into this majority category with a mean score of 533 (Thomson et al., 2020). A score of below 550 indicates that a student would have difficulty generalising their science skills and knowledge to novel contexts. Research into the higher performing nations, including Korea, Singapore, Japan, and Finland, highlights complex cultural, social, and political differences that may not be readily applicable to lower performing nations (Federick, 2020; Kaya & Rice, 2010; Lee & Kim, 2019; Yamanaka & Suzuki, 2020). For example, Hierarchical Linear Modelling analysing educational variables across five nations (including Japan and Singapore) showed that higher home resources and parental self-confidence were more closely associated with performance than instructional variables (Kaya & Rice, 2010). Cultural respect for teachers and multi-decade programs of educational reform may also contribute to higher performance (Federick, 2020; Lee & Kim, 2019; Yamanaka & Suzuki, 2020). However, these factors are contextually bound and require large scale collaboration and investment to change. Lower performing nations are likely to have unique enabling factors that could serve as a basis for long-term improvement. For example, primary students in Australia and abroad still express curiosity for and interest in their science learning (ACARA, 2013, 2019; NASEM, 2022) in ways could bode well for future development.

To improve science outcomes by capitalising on young peoples’ scientific curiosity, a mature evidence base of student-centred approaches now offers guidance for the provision of high-quality science teaching education experiences in primary classrooms (Aubusson et al., 2015, 2019; Deehan et al., 2022; Skamp & Preston, 2021). Indeed, a recent scoping review presented evidence that student-centred approaches, such as: inquiry learning, cooperative learning, community engagement and nature of science instruction have been associated with large to very large effect size improvements in primary students’ science content knowledge, skills and attitudes (Deehan et al., 2022). In fact, some science interventions have reported effect size increases of greater than two for participants’ science content knowledge (e.g.Ocak, 2010; Panasan & Nuangchalerm, 2010), skills (e.g.Akcay & Yager, 2010; Safaruddin et al., 2020) and attitudes (e.g. Prokop et al., 2007); evidence that some within the field appear to be solving the 2-sigma problem (i.e., the educational challenge of approximating the impact of 1-to-1 tutoring in typical classroom settings) within specific contexts, which leads to more equitable improvement for learners (Bloom, 1984). For educators, Harlen’s (2015) big ideas and Roth’s (2014) attributes of effective primary science teaching can further contextualise student-centred science teaching practices.

However, the concept of “best practice” will always be context specific and based on the professional judgements of individual educators. Long-term, sustained improvement to the quality of science education is still inhibited despite the ever-strengthening evidence base; this is, in large part, due to the social and contextual nature of education as student characteristics, school circumstances, curriculum demands, teacher traits and societal pressures make a universal definition of “best practice” impractical. Notions of “best practice” in the choice of teaching strategies are often subverted by workload and professional stressors. For example, a study of the workload and stress levels of 749 Australian teachers showed that more than half of respondents were very or extremely stressed and were considering leaving the profession due to workload and well-being issues (Carroll et al., 2022). In essence, primary science education is hindered by long-standing theory-praxis divides (Anagnostopoulos et al., 2007; Deehan et al., 2020; Holbert et al., 2011) that are driven by systemic challenges.

The challenge of bridging the theory-praxis divide in science education is exacerbated for teachers by systemic issues, including unmanageable workloads (Johari et al., 2018; Kokkinos, 2007), disruptive student behaviours (De Nobile et al., 2015, 2016), overwhelming curricular demands (Crump, 2005; Jenkinson & Benson, 2010), and resourcing inadequacies (Gonski, 2011; Rowe & Perry, 2020). Recent shifts in global science curricular to inquiry models (ACARA, 2017; Bybee, 2014; Eggleston, 2018; Kim et al., 2013; NGSS, 2013), although defensible in terms of scholarly evidence, require teachers to implement more time and resource intensive approaches, often without adequate structural assistance (Akuma & Callaghan, 2019). A systematic review of 29 research articles found that teacher confidence, limited equipment, adjusting existing learning progressions, and student disengagement all present considerable challenges to inquiry teaching in science classrooms that serve to exacerbate other systemic challenges (Akuma & Callaghan, 2019). Also, despite recent reports of primary teachers engaging their students through student-centred and active science teaching approaches (ACARA, 2013, 2019; Banilower et al., 2018; Banilower, 2019), teachers can have limited science content knowledge and interest (Appleton, 1992, 2003; Deehan, 2022; Murphy & Smith, 2012) which can diminish the quality of science education (Goodrum & Rennie, 2007; Watters, 2014). Research has shown that primary teachers often hold similar alternative science conceptions to primary learners, including the false belief that the absence of motion denotes the absence of force (Narjaikaew, 2013) and incorrectly attributing the Earth’s magnetic field to gravity or its revolution around the Sun (Dahl et al., 2005). Additionally, primary science education can be adversely impacted by inadequate curriculum time (Angus, 2003; Goodrum et al., 2001; Office of the Chief Scientist, 2012; Tytler & Griffiths, 2003; Tytler et al., 2008) and overreliance on disengaging pedagogies (Goodrum & Rennie, 2007; Goodrum et al., 2001; Tytler et al., 2008).

Clearly, more needs to be done to translate the expansive evidence base of primary science teaching practices into improved teaching practice and student outcomes. Although Initial Teacher Education (ITE) programs have made promising strides in adapting their primary science programs to reflect inquiry-based and student-centred science teaching (Deehan, 2021, 2022; Fitzgerald et al., 2021), the university sector does not reflect the unique and pressing challenges of the primary education sector. Accordingly, this paper seeks to draw on the perspectives of current Australian primary teachers about how they believe primary science education can be improved. As a group, primary teachers should be the focus of ongoing improvement efforts as they are directly responsible for bridging theory-praxis divides. Furthermore, there is evidence suggesting that many primary teachers are willing science teachers who seek to actively engage their learners through student-centred approaches (ACARA, 2013, 2019; Banilower et al., 2018; Banilower, 2019). Many of the scholarly literature reviews examining the impact of student-centred practices are testaments to primary teachers’ ability to support students’ science learning (Aubusson et al., 2015, 2019; Deehan et al., 2022). Even early career teachers have displayed a willingness to function as science agents of change by assuming leadership roles to overcome systemic challenges (Deehan et al., 2020). The current study is an overdue contribution as much of the keystone work in this space is around 20 years old (e.g., Goodrum & Rennie, 2007; Goodrum et al., 2001), meaning that the voices of the current generation of primary educators have not been widely heard in primary science education research and public discourse. The following research question will be answered in this paper:

- How does a sample of Australian primary teachers believe the quality of Australian primary science education could be improved?

## Methodology

Australian primary teachers employed in a public jurisdiction were asked how primary science education could be improved via an online survey. The question was open-ended in its framing. The qualitative design of this project accords with the interpretivist and constructionist perspectives that underpin the literature review and research question (Bryman, 2016; Clark et al., 2021). The interpretivist epistemological orientation reflects the underlying assumption that individuals’ perceptions of their social worlds influence actions and results. The constructionist ontological perspectives acknowledges that teachers, as individuals, influence the institutions within which they work. Ethics clearance was gained from the university Human Research Ethics Committee (HREC) (H21071) and the targeted school jurisdiction (2021178).

### Recruitment and sample

The context of this study was a jurisdiction of approximately 30,000 educators employed across over 1600 Australian primary schools. Non-probabilistic convenience sampling strategies were employed, including mail outs to schools, email invitations to principals, social media sharing, snowballing and dissemination across relevant professional networks. Prize draws of gift cards and science teaching resource vouchers were used to incentivise participation. Participants were recruited from July through to November 2021.

One hundred and sixty-five Australian primary educators from 150 schools participated in the research project. The demographics of the sample are presented in Table 2, including gender, age, employment status, school location, years of experience and school socio-economic status. Although this project can make no claim to generalisability, it is worth noting that the sampling ratio (1:188) compares favourably to the last national investigation into Australian science education (1:400) (Goodrum et al., 2001). Furthermore, one out of every 11 schools in the targeted jurisdiction are represented in the sample.

### Improvement question survey

Participants responded to a digital survey through direct hyperlinks and QR codes. After providing informed consent, participants were invited to provide their demographic data (see Table 1). The following question was then presented, *How do you think the quality of primary science education could be improved?*

**Table 1:** Participant demographic data (*N* = 165)

Gender	
Female	Male
131	34

Age					
18–24	25–34	35–44	45–54	55–64	65 +
2	47	40	48	24	4

Employment status		
Full time	Fixed term contract	Casual
106	55	4

School location*		
Metropolitan	Non-Metropolitan	Unspecified
73	87	5

Averages		
Years of experience	School ICSEA**	School ICSEA %
15.8	983.27	41.57

*School Location is based on the Australian Statistical Geographical Standard (ABS, 2016)

**ICSEA Index of Community Socio-Educational Advantage (ACARA, 2016)

### Data analyses

To uphold the foundational principles of qualitative research (Glaser & Strauss, 1967), the open, axial, and selective coding phases were collaborative and iterative (Williams & Moser, 2019). The nonlinear, reflexive approach to data analysis accorded with the constructionist and interpretivist perspectives inherent to the research question. The early open coding phase was characterised by unstructured researcher immersion in the qualitative data; a core condition necessary for effective emergent coding (Bryman, 2016; Clark et al., 2021; Strauss & Corbin, 1990). Axial coding commenced as the researchers began to pose themes and group participant responses accordingly using QSR NVIVO 12. The selective coding phase was characterised by the refinement of the themes to ensure the most concise and accurate representation of the participants’ views. Researcher bias was mitigated in the selective coding phase as the number of respondents contributing to each theme was used as an indicator of relative thematic prominence.

The trustworthiness and credibility of the data analyses were strengthened by consistent collaboration and reflection across all phases. Interrater reliability was achieved through collaborative consensus on the coding of all responses. The research team’s coding was also checked by an external research assistant for coding errors and alternative choices; the research assistant affirmed the research team’s analyses. Jaccard’s Similarity Coefficients (Krebs, 2014), a measure of response overlaps amongst codes on a zero (no similarity) to one (duplication) scale, was used to identify redundant themes. No theme pairings produced a similarity coefficient of greater than 0.2, indicating that themes are sufficiently delineated. However, the lack of overlap may also be attributed to the asynchronous data collection method wherein participants were not afforded the chance to cover multiple themes in extended dialogues.

### Results: how does a sample of Australian primary teachers believe the quality of Australian primary science education could be improved?

The sampled primary educators offered an array of ideas and perspectives on how Australian primary science education practice could be improved. Table 2 provides source counts, percentages, and illustrative quotes for each of the 17 themes which emerged from the qualitative data analyses. Nearly half (47.27%) of all the responses cited the need for more science professional development opportunities. One quote captured the general sentiment of the respondents, *I think there needs to be more Professional Learning for teachers on how to create relevant, fun and engaging lessons!,* as many made general, unspecific requests for PD opportunities. Another felt that PD could ensure primary teachers *don’t shy away from teaching science as they don’t feel confident enough*. The more specific PD suggestions, such as: *by teachers for teachers*, *teaching the science concepts*, *explore effective pedagogical practices*, *on-demand, online PL*, and *NOT online*, highlight the varied science PD needs of the respondents. Perhaps unsurprisingly, over a third (37.58%) of respondents believed improved science resourcing and funding would improve the quality of primary science education. Recommendations for *examples of possible programs*, *access to resources at a national level*, *sites where students can log data*, *a dedicated science room*, and *videos for teachers* were present within this theme. Two quotes suggest that teachers cover resourcing limitations themselves, *I currently spend approximately $20 a week* and *teachers should not have to purchase their own supplies!!!!*; both are indicative of more generalised educational threats of teacher payrates and funding.

**Table 2:** Improvement in primary science education

Theme	Number of contributing sources	Percentage of codes (318)	Percentage of respondents (165)	Illustrative quote
Professional Development	78	24.53	47.27	*I believe further professional development would be a huge asset.*
Funding-resources	62	19.50	37.58	*More resources provided to school, so teachers are not constantly reaching into their pockets to do hands on activities and experiments.*
Classroom practice	36	11.32	21.82	*Teachers need to teach science in engaging ways, using technology and effective practice.”*
Personal-teacher improvement	35	11.01	21.21	*Teachers often fear science and say they can’t teach it.*
Time	22	6.92	13.33	*I only teach science and robotics across all year levels. I feel like I am always ON and setting up, shopping, planning, and teaching *etc.*is full on. I’m so tired! I’d love an assistant for’ couple of ho’rs a week!* *OR* *Time – always seems to be a rush and can never investigate topics as thoroughly as needed.*
Cross curricular integration	19	5.97	11.52	*Science needs to be integrated into English and maths KLAS to give it more time in the week”*
Curriculum	15	4.72	9.09	*The upper primary NSW science syllabus is very dense and trying to teach all content of all four strands in one year at quality level is not possible*
Specialists	10	3.14	6.06	*Having specialist science teachers in primary school*
Collaboration (mentors-team teaching-PLNs*)	10	3.14	6.06	*Continued collaboration between staff*
Partnerships (University-Others)	9	2.83	5.45	*Connections with real STEM professionals to break stereotypes of what it is to work in these fields and who can do it.*
Technologies	6	1.89	3.64	*Ideas on how to integrate digital, design technologies into teachers’ timetables.*
Leadership	5	1.57	3.03	*Support from curriculum leaders.*
pre-service education	4	1.26	2.42	*Increase the quality of training in University*
Specific approach or resource	4	1.26	2.42	*I follow the Inquisitive.com lessons and adjust them as necessary.*
State assessment	1	0.31	0.61	*Set state assessments (like Maths and English) to compare where students are.*
Smaller class Sizes	1	0.31	0.61	*Smaller classes for science*
Indigenous science	1	0.31	0.61	*More time allowed for PD, particularly in the areas of Indigenous Science and the use of certain Technology*

*PLN Professional Learning Networks

The third (Classroom Practice) and fourth (Personal-Teacher Improvement-Limitations) most prominent improvement themes, both accounting for approximately 21% of responses, suggest that a portion of teachers feel they and their peers are also responsible for improving the quality of primary science education. Calls for improved classroom practice align broadly with evidence-based practices, including “*inquiry learning*”, “*hands on learning*”, and “*real life experience*”, with these and the more general calls for more engaging teaching being linked to other recommendations. Many respondents believed that improved practice would require both personal, *I feel I don’t have the knowledge of science processes and content to teach it well*, and profession wide improvement, *Teachers often fear science and say they can’t teach it*. Clearly, a significant portion of respondents feel that educator improvement must occur alongside systemic support.

Themes of Time (13.33%), Cross Curricular Integration (11.52%), Curriculum (9.09%) and Specialists (6.06%) indicate that some of the respondents believe there are possible ways of adjusting the position of science in the primary science curriculum for the sake of improvement. Several respondents felt there was insufficient time to adequately teach science, as one respondent lamented that there, *always seems to be a rush and can never investigate topics as thoroughly as needed*. One teacher espoused their beliefs about the role of cross curriculum integration in addressing the time issue, *More integrated teaching approaches. Science needs to be integrated into English and maths KLAs [Key Learning Areas] to give it more time in the week.* Among the critiques of the curriculum the issue of overcrowding emerged, *less extensive syllabus which I think limits deep learning and thinking*. The opposite position was also evident as some respondents advocated for *specialist science teachers*, rather than generalist primary teachers.

Several themes were notable for their limited prominence amongst the improvement themes. The relative lack of advocacy for more informal collaborations (including mentors, team teaching & PLNs) (6.06%) and institutional partnerships (5.04%) indicate that many participants do not view universities as vital stakeholders in advancing primary science education. However, one educator felt the university sector was important, *I loved my science units (2) at university, which was purely because of the knowledge the lecturer had in the KLA of science. I believe that more of these lecturers need to be utilised by universities to educate and engage us more deeply with the teaching and (our own personal) learning of science*. In an extension of this point, only five educators identified the need for improvement or change in school-based leadership (3.03%). Also of note was that some traditional mainstays in the science education and general education literature, such as pre-service teacher education (2.42%), State Assessment (0.61%), Smaller Class Sizes (0.61%) and Indigenous Science (0.61%), were comparatively underrepresented in the perspectives of this sample of primary educators.

## Discussion

In answering the research question (*How does a sample of Australian primary teachers believe the quality of Australian primary science education could be improved?*), the themes derived from the respondents’ qualitative data emphasised teachers as being central figures in the improvement of Australia primary science education. Three of the four most prominent themes show this view that internal improvement by teachers is key (Professional Development, Classroom Practice & Personal-Teacher Improvement). Professional Development (PD) was seen by the sample of primary educators as the most important factor in improving the quality of Australian primary science education. Indeed, just under half (47.27%) of the 165 primary educators identified PD as a point of need. Access to science PD could also address the needs for enhanced classroom practice (21.82%) and general teacher improvement (21.21%) mentioned by a substantial minority of respondents. This is a case where the divide between teachers and academics (Anagnostopoulos et al., 2007) may have been bridged as the traditionally limited body of science PD evidence (Scher & O’Reilly, 2009) now contains strong evidence of the positive impacts science PD programs can have on teachers and students alike (Deehan, 2017; Kleickmann et al., 2016; Lynch et al., 2019; Yoon et al., 2007). Australia has been a leader in science PD provision and evaluation in recent years, with both formal and informal science PD programs being established across different modes and jurisdictions (Aubusson et al., 2019; Hume, 2012; Ng et al., 2018; Ralls et al., 2020; Stevenson et al., 2019). This is a fortuitous finding as it shows that there may be an authentic need for science PD emerging in school contexts that can be supported by established and emergent programs.

Although there can be no universal definition of “best practice” in primary science PD, we do know that evidence-based, job-embedded PD results in positive outcomes for learners. Mutch-Jones and others (2022) used multilevel modelling on data obtained from 1546 classrooms to examine the impact of teachers, including PD engagement, on students’ scores on a state science assessment. They found that the students of teachers who had participated in a collaborative, hands-on, long-term science PD program had significantly higher science achievement scores than comparison groups. A recent survey of 171 Australian educators provides greater insight into the preferred characteristics of science PD, with the sample greatly favouring PD focusing on investigation, inquiry, and science teaching strategies (Burke et al., 2022). By contrast, argument-based science, collaboration, and Professional Learning Networks (PLNs) were deemed least important by the teachers. Stakeholders should now be focused on facilitating as much science PD for willing participants as possible in ways that respond to teachers’ needs and expand upon and reflect the scholarly evidence base (Mutch-Jones et al., 2022). Clearly, a greater array of professional development programs, with varied structures and foci could be valuable in aiding what appears to be a receptive group of primary educators.

Despite the internal focus of many teachers in the sample, the ever-present issue of inadequate resourcing and funding was identified as an area of improvement by over a third of the respondents (37.58%). Given that the participating teachers were from an Australian public school system, the theme of inadequate resourcing, both material and financial, is likely related to the long-standing problems of funding equity and resource inadequacy across Australian schools (Gonski, 2011; Rowe & Perry, 2020; Thompson et al., 2019). It should also be noted that these risks exacerbate existing socio-economic and geolocation divides (Fraser et al., 2019; Sullivan et al., 2018). Funding and resourcing issues represent a particularly strong threat to any efforts to improve the quality of Australian primary science education. This means that all stakeholders, whether they be teachers, administrators, academics, etc., need to publicly argue for expanded investment in primary science education to maximise the benefits of engaging an increasingly willing base of teachers with established and emerging support mechanisms.

The absence of mainstay themes in education research within participants’ responses was also noteworthy as Time (e.g., AITSL, 2021; Crump, 2005; Jenkinson & Benson, 2010), Cross Curricular Integration (e.g., Gresnigt et al., 2014; Rennie et al., 2018), Specialist Science Teachers (e.g., Ardzejewska et al., 2010; Levy et al., 2016; Mills et al., 2020; Roach & Wendt, 2022), School-University Partnerships (e.g., Hobbs et al., 2018; Kenny et al., 2014) and Preservice Science Education (Deehan, 2017, 2021, 2022) were not widely identified as key factors in the ongoing improvement of Australian primary science education. While a sampling error or methodological limitations could explain the relative paucity of these themes, they could also be an extension of the previously mentioned internal locus of control expressed by teachers as they view themselves, rather than universities or specialist teachers, as being central to the improvement of primary science education. This is particularly positive given that the educational jurisdiction from which the participants were drawn is renowned for exceptionally high teacher workloads (AITSL, 2021) and a reliance on outsourcing science from generalist to specialist teachers (Ardzejewska et al., 2010). Curiously, despite the historic divide between schools and universities (Anagnostopoulos et al., 2007; Deehan et al., 2020; Holbert et al., 2011), universities, academics and initial teacher education did not feature prominently as either hindrances or benefits to Australian primary science education. This may suggest that teachers are not aware of the role that universities could play in supporting primary science teachers, which is unsurprising because most preservice primary science PD programs, such as Primary Connections, are offered by separate government or private institutions. Perhaps universities could extend their strong primary science initial teacher education programs (Deehan, 2021, 2022; Fitzgerald et al., 2021) into accessible micro credentials that provide teachers with the support they desire whilst maintaining the long-term viability of university ITE programs in the wake of the Covid-19 era (e.g., Gore et al., 2021).

There are sampling and methodological limitations to consider in any interpretation of the findings. Although the sampling ratios achieved for both participants (1:188) and schools (1:11) are relatively strong in the context of Australian science education research (Goodrum et al., 2001), they still fall well below the 30–35% sampling rates typically sought in general survey-based research (Nulty, 2008). Additionally, the absence of a probabilistic sampling frame limits the generalisability of the findings as there are likely to be members of the target population who may have elected to participate but were not afforded the opportunity. This means that there is a “silent voice” threat to this research as there are many members of the target population who did not participate, either due to a lack of awareness or will. It is very likely that the sampled teachers are more confident and engaged science educators than many non-respondents, and this may explain the prevalence of internally focused themes. The asynchronous survey methodology, despite its benefits in terms of accessibility, cost effectiveness and social desirability bias mitigation, is likely to have limited the depth of the qualitative data collected. While the open structure of the qualitative question has prevented responses from being limited by the researchers’ conceptual framing, the asynchronous method prevented further detail from being elicited from participants. For example, while many participants explicitly cited a need for more science PD to improve Australian primary science education, proportionally few articulated modes, topic areas or types of PD they may desire. If anything, this means that the research should ideally serve as a catalyst for productive engagements between schools and other stakeholders. Indeed, if teachers’ advice about improving primary science education is to be acted upon, more research and/or consultation is needed to understand their professional education journeys and school contexts.

The findings presented in this paper give rise to some tentative implications for researchers and other educational stakeholders. The limited scope of this study could be addressed by multi-jurisdictional, national and/or international research into primary science education. Additionally, the reported desire for internal teacher improvement, primarily through science PD opportunities, suggests that it is worthwhile to continue to investigate and remediate barriers to primary science PD access. Deeper research into primary teachers’ experiences of science PD, hindering factors, preferences and engagement rates would be valuable given that Australian PD mandates for accreditation are, by necessity, very broad. In fact, no Australian jurisdiction has any minimum requirement for the science PD of primary teachers (ACTTQI, 2022; AITSL, 2017; NESA, 2017; QCT, 2022; TRBNT, 2022; TRBSA, 2022; TRBT, 2022; TRBWA, 2022; VIT, 2022). Universities could expand their roles in researching and providing science PD opportunities and support to primary science education through structured PD programs (Aubusson et al., 2019; Hume, 2012), Professional Learning Networks (Ralls et al., 2020; Stevenson et al., 2019) and short-form micro credentials (Miller & Jorre de St Jorrre, 2022; West et al., 2020). In a macro-sense, such initiatives would be mutually beneficial ways of offering support where need has been identified in ways that bridge historic school-university divides (Anagnostopoulos et al., 2007; Deehan et al., 2020; Holbert et al., 2011).

## Conclusion

This paper has drawn on the perspectives of Australian primary teachers to better understand the theory-praxis divide in primary science education. Views regarding the improvement of primary science education were understandably diverse amongst the sample of 165 Australian primary science educators. However, as a broad group the teachers viewed themselves and their peers as central to improvement and believed they should be supported through access to professional development and better resourcing. The teachers’ sense of agency should be fostered through support and ongoing engagement. The research presented in this paper should serve as both a catalyst for extended research and a justification for expanded professional development opportunities and financial support. Indeed, there appears to be an observable synergy between the improvement beliefs of teachers and the existing strengths of universities and professional development providers in supporting teachers.
